# Mr. Scott, on the Medicinal Effects of Cobweb

**Published:** 1809-11

**Authors:** James Scott

**Affiliations:** Surgeon H. M. S. Euryalus, off Dungeness


### *To the Editors of the Medical and Physical Journal.*

> "Who shall decide, when Doctors disagree,
> "And soundest casuists doubt, like you and me?" POPE.

GENTLEMEN,

IN my Paper on the Virtues of Cobweb, (in No. 127 of your Journal) I promised to take the earliest opportunity of exhibiting that substance, and am happy to say, that I have

I have lately witnessed its good effects in a case of tertian ague, under my own care.

Andrew Yeaman, ætat. 19, trumpeter in the Staff-Corps, was embarked on board this ship in the late Expedition against Flushing, and had laboured under intermitting fever nearly a month, by which he was much emaciated, and reduced to an alarming state of debility. He had been stationed at Hythe, where I understand intermittents are very prevalent, (in consequence, it is believed, of the vicinity of the Royal Military Canal), and by his own account had taken large quantities of bark with wine and opium. Those medicines had never, in the slightest degree, shortened the duration, nor diminished the severity of the paroxysms, and seemed to have produced no other effect than, probably, that of preventing the fever from degenerating into a more continued type, or changing its period of accession. The intermissions, in this case, were most distinct, and unattended with any symptoms indicating disease, except extreme weakness, and a more ghastly paleness of the countenance than I had ever witnessed.

As I was most anxious to give the cobweb a fair trial, I requested my assistant (Mr. Farley) to be particularly careful in observing the patient's state and the effects of the remedy, both which he watched with the utmost assiduity; and I feel much pleasure in adding his testimony in behalf of its utility. A quantity of cobweb, not exceeding eight or nine grains, was gathered from the bread-room, and made into two pills; one, consisting of five grains, with sufficient mucilage of gum arabic to form it; and the other, of the remaining cobweb, prepared in a similar manner. The five grain pill was given on an empty stomach, a little after eight o'clock in the morning, (being two hours previous to the expected period of accession), and the other at the usual hour of the paroxysm, which was not felt at all. It may be proper to state, that no other medicine was given with the cobweb; and it will appear, that he did not take nearly the quantity prescribed by Dr. Jackson, as the ship was at sea, and I could not procure more than I have mentioned. The paroxysm never returned; but a slight rigor was felt about the second period from the exhibition of the cobweb, and easily yielded to two grains of opium, followed by bark and sulphuric acid, which completed the cure.—As far as I could learn, arsenic had not been resorted to on this occasion.

The "Essex Practitioner" and I, seem to entertain diametrically opposite opinions respecting the operation of the spider which was swallowed by the lady; and he thinks that case "will go but little way to corroborate Dr. Jackson's testimony." With him, in all probability, it will go no way at all, because he appears to be already convinced of the inutility of the substance in question; but with many others, it is to be hoped, it may have some little weight; and at any rate, it cannot in the least invalidate Dr. Jackson's high opinion of the sanative powers of spider's web. We need not be told, that fear "will produce even death itself;" but it requires no small degree of faith to believe that the slight degree of that passion, or rather of disgust, which would be excited by swallowing a spider, can at once dissever the strong chain of a confirmed disease. It is a melancholy proof of the imperfection of the medical art, that its professors are sometimes obliged to attribute double, and even opposite, virtues to certain remedies. In my opinion, however, no medicine can have more than one mode of action, but attending circumstances may often alter its apparent effects; and I believe the credit which has been given to the supposed sanative virtues of fear, are more justly due to concomitant accidents.

Every one who has practised in climates unfavourable to European constitutions, must have seen the bad effects of fear in those countries; and almost every one will agree, that numbers, who might otherwise have escaped tropical diseases, have been prematurely hurried into Eternity by apprehension of death; whereas, others, constitutionally predisposed to disease, have been saved by that inestimable cordial—Hope. When a drunken man falls into the water, and is thereby instantly restored to a state of sobriety, (as I have frequently seen) how is his recovery to be accounted for? Is it to be attributed to fear? No: at the time of his immersion, neither fear nor any other passion agitated his bosom; before the accident he was in a state of exhaustion, from the inordinate stimulus of the liquor; he was, perhaps, on the verge of typhus fever; but when by chance he was plunged into the salutary bath, the action of the heart and large arteries became increased; and unless he suffered an extreme degree of cold, no febrile affection succeeded. In such an instance, therefore, fear cannot be concerned.

I know perfectly well, that authors of high celebrity have mentioned fear as one of the means of curing intermittents; tents; but what they term fear, I would call disgust; for it is well known that many people who are cured by bark and opium, have as great an aversion to those drugs, as to spiders or toads; yet the medicines succeed, because they really possess medicinal virtues.

In all probability, the "Essex Practitioner" has used tourniquets in intermittents, (as recommended, I believe, by Mr. Kellie, a naval surgeon) or, if he has not, and will give even a moderate degree of credit to their beneficial effects in the hands of others, I doubt not he will agree that in such cases the cure is effected, according to the suggestion of the ingenious proposer, by the tourniquet preventing the circulation in the extremities to which they are applied, and of course, "causing a more speedy and abundant return of blood to the heart." Exactly so do I conceive the cold water to operate on the drunken man, by diminishing the morbid heat and increased action of the extreme vessels; and the same is observable after amputation of a limb, or when any bulky part of the body is removed, unless much hæmorrhage occur during the operation; for the blood which used to circulate in the member which suffers amputation, remains in the body, and on that account, in robust constitutions, copious venesection is very generally required. I would not have it supposed from what I said in my last paper, that I doubt the veracity of the anonymous class of writers in general, but I must still think that the "Essex Practitioner's" arguments in favour of such signatures as he uses, are by no means conclusive; and I beg to apprize him of his mistake, in asserting that I may administer my medicines "in what quantity and form I please, with impunity;" nor can I see why the "constitution of a sailor will hardly bear a comparison with that of a weak delicate female."

The complaints of the former are, it is true, very generally of an acute nature, and less complicated than those of the fair sex; but in the decline of life, and when their vigour has yielded to laborious duties, there is no species of nervous complaint from which sailors are exempt; and we daily see among them hypochondriasis and hysteria, with all the miserable symptoms which characterize those diseases.

If he were better acquainted with the medical department of the Navy, he would be convinced that the practice of Naval Surgeons is "watched with as unceasing attention" as that of any Country Practitioner, and perhaps, with more prying and laudable curiosity. Their embar-

 rassments rassments must also be greater; for in the hour of difficulty and danger, the Naval Surgeon is most frequently obliged to form a prompt decision; too often he has no professional friend to consult with; and labours under many disadvantages, which will be readily conceived and acknowledged by generous minds, although I blush to say that some members of the medical profession on shore and in the land-service, have disgraced themselves by casting very unwarrantable reflections on a numerous body of men, by no means their inferiors in point of education, and, I trust, equally faithful and conscientious in the discharge of their duty.

On the contrary, a Country Practitioner can in all difficult cases, have easy recourse to the advice and assistance of his brethren; nor will the unfortunate issue of any case reflect dishonour on him, more than on the Naval Surgeon, if the evil should be supposed to arise from any novelty or rashness in the mode of treatment. In the event of such a suspicion attaching to the character of a Naval Surgeon, his pecuniary interests would not perhaps be so immediately affected; but that character on which he must ground his hopes of future advancement, would undoubtedly suffer in the opinion of those who dispense the naval medical honours, and of course his fortune even, would be eventually ruined thereby.

The Country Surgeon is not, I believe, obliged to give an account of his practice; but the Naval Practitioner is required to keep a very strict Journal, which must be submitted to the inspection, and obtain the sanction, of a medical board, before he can receive his pay. I further conceive it a part, and it is a very pleasant part, of my duty on this occasion, to mention that care, tenderness, and generosity which are extended to sick sailors, by their officers, who are by no means inattentive to the Surgeon's conduct; and it will be readily admitted that men of sound understanding, frequently adorned with liberal education, who associate with him, cannot be very incompetent judges of his professional abilities. Indeed, there are very few officers who have not some medical knowledge, naturally arising out of their intimacy with the surgeon, and that praise-worthy curiosity which I hinted at above; and I know several who have made very considerable acquirements in that way.

I must here beg your indulgence in allowing me to state, on the authority of a gentleman, a particular friend of mine, an instance in which cobweb has been peculiarly serviceable; serviceable; and although I feel that I have almost already transgressed the bounds within which I ought to confine my paper, yet I trust the nature of my subject, and the few opportunities I may enjoy to communicate with you, will plead my excuse, and gain it admission into your most valuable Journal. Walter Sands, Esq. purser of his Majesty's ship Camilla, has been afflicted for many years with a very distressing asthma, which has proved fatal to his father, and called to an early grave two beautiful and accomplished women, his sisters. As the complaint appears to be hereditary, and in this gentleman to be aggravated by mal-formation of the thorax, no remedies gave any permanent relief, nor did change of climate procure any alleviation of symptoms. He has taken, at various times, under the direction of eminent physicians, the fox-glove in every form, with all other medicines which have been recommended; and frequently in a kind of despair has resorted to almost every empirical composition from which he could expect advantage; but in vain.

For a considerable time back he has never been able to lie down in his bed, on account of a sense of suffocation, which he always experiences in a horizontal posture, but is obliged to be supported half-sitting, by pillows, and even then is tormented to such a degree by incessant cough, so violent as often to induce vomiting, that he can seldom sleep before two or three o'clock in the morning, and his slumbers are often interrupted. About three weeks ago I was witness to his sufferings, which came on, on board this ship, after he had been dining with me, and I much regretted that I had no cobweb to administer, as I was well aware of the inefficacy of all other medicines in his case. I however mentioned the subject to him, and the following day he collected nearly a scruple of the spider's web, which he swallowed at bed-time, and to his utter astonishment, enjoyed sound and uninterrupted sleep all night; a blessing, to which he had been an entire stranger above six years. Since he began the cobweb, he thinks his health is improved; the cough has certainly abated; but he finds much difficulty in procuring a sufficient quantity of the web, and whenever it is omitted the complaint returns. Your suggestion respecting the different sorts of cobweb, appears rational, and those who have an opportunity to make experiments, will discover what species or variety of spider produces the most efficacious web. There are two kinds of those insects, common in cellars and moist places; one has a round body, brown speckled back,

 and and very short legs, which easily distinguish it from the other, whose body is oblong, and of a pale greyish cast, with very long and semipellucid legs. The web of the former has a very compact texture, and a rich bombycinous appearance; that of the other is much less compact, resembling a net in some degree; has no sensible taste nor odour (both which qualities are slightly perceptible in the other), and is destitute of that rich silk-like appearance which characterizes the first mentioned species.

If these few observations at all assist in drawing the attention of the Faculty to a subject which, I trust, would amply reward their labours, I shall be extremely happy, and my humble endeavours shall not be relaxed until I have clearly demonstrated the medicinal qualities of cobweb, or till I shall find by experience, that it does not possess the virtues which have been ascribed to it. I must further beg your indulgence, and that of the medical public, for the inelegant style of this letter, as I did not see your two last Numbers till within a very few days, on my return from the Scheldt; and as I am about to proceed to a foreign station, I am anxious that it should obtain an insertion in the next Journal, if possible, on which account, I venture to send it into the world "with all its imperfections on its head."

I am, &c.

JAMES SCOTT, Surgeon

*H. M. S. Euryalus, off Dungeness.*

*Sept.* 18, 1809.

